# Laparoscopic management of a pediatric pantaloon hernia with contralateral patent processus vaginalis: a case report

**DOI:** 10.3389/fped.2026.1941318

**Published:** 2026-09-01

**Authors:** Huinan Wang, Dongsheng Li, Ye Yao, Yang Zhang, Jinsong Tang

**Affiliations:** Department of General Surgery, Northern Jiangsu People's Hospital, Yangzhou, Jiangsu, China

**Keywords:** child, inguinal hernia, laparoscopy, metachronous hernia, pantaloon hernia

## Abstract

Pediatric direct inguinal hernia is a rare entity, and the coexistence of ipsilateral indirect and direct inguinal hernias (pantaloon hernia) is even more uncommon. Consequently, pediatric surgeons have limited experience with this entity. We report a 12-year-old boy who presented with a reducible right inguinal mass. Laparoscopy revealed a right-sided pantaloon hernia (predominantly indirect) and a contralateral patent processus vaginalis on the left. We performed transumbilical single-port laparoscopic bilateral high ligation of the hernia sacs (including both the direct and indirect components on the right) combined with reinforcement using the right medial umbilical ligament. The procedure was uneventful, and the patient was discharged on postoperative day 1. At 6-month follow-up, no recurrence, pain, or groin infection were observed, and the cosmetic outcome was excellent with minimal scarring. We believe that laparoscopy offers unique advantages in pediatric inguinal hernia repair by enabling accurate identification of “atypical” hernias, reducing missed diagnoses, and allowing contralateral exploration to decrease the risk of metachronous hernias, thereby minimizing unnecessary surgical and anesthetic risks as well as financial burden. We share our experience to provide an alternative therapeutic option.

## Introduction

Inguinal hernia is a common condition in pediatric surgery, with indirect hernias being the most frequent type. Direct inguinal hernias are relatively uncommon, accounting for approximately 2% to 3% of all pediatric inguinal hernias (1, 2). The coexistence of both direct and indirect hernias on the same side (pantaloon hernia) is even rarer (1, 3). Laparoscopic repair offers several advantages, including smaller and more aesthetic incisions, lower risk of postoperative infection, less pain, shorter operative time, and faster recovery (4, 5). Moreover, laparoscopy provides a clear and detailed view of the inguinal region, facilitating the detection of rare hernias that are often missed during open surgery, such as direct, pantaloon, and femoral hernias (4, 6). It also enables evaluation of the contralateral groin for occult hernias (5, 7–9); when bilateral patency is present, simultaneous high ligation of both hernia sacs (or processus vaginalis) can be performed without significantly prolonging operative or anesthetic time. We report a 12-year-old boy in whom laparoscopy revealed a right pantaloon hernia and a left patent processus vaginalis, both successfully managed in a single procedure with satisfactory results.

## Case presentation

A 12-year-old boy presented with a 10-day history of a reducible right inguinal mass. The swelling was more pronounced after prolonged standing or physical activity and subsided with recumbent rest. There was no history of incarceration. Physical examination revealed a soft, reducible mass in the right inguinal region, measuring approximately 3 cm × 4 cm × 2 cm, which did not extend into the scrotum. Preoperative ultrasonography suggested a right inguinal hernia containing bowel loops. The patient was born at full term and currently weighs 34 kg. He had no history of chronic cough, constipation, connective tissue disorders, or prior surgical interventions, and there was no family history of inguinal hernia. After routine preoperative workup was completed, the patient was scheduled for same-day surgery.

### Surgical procedure

**Figure 1:**
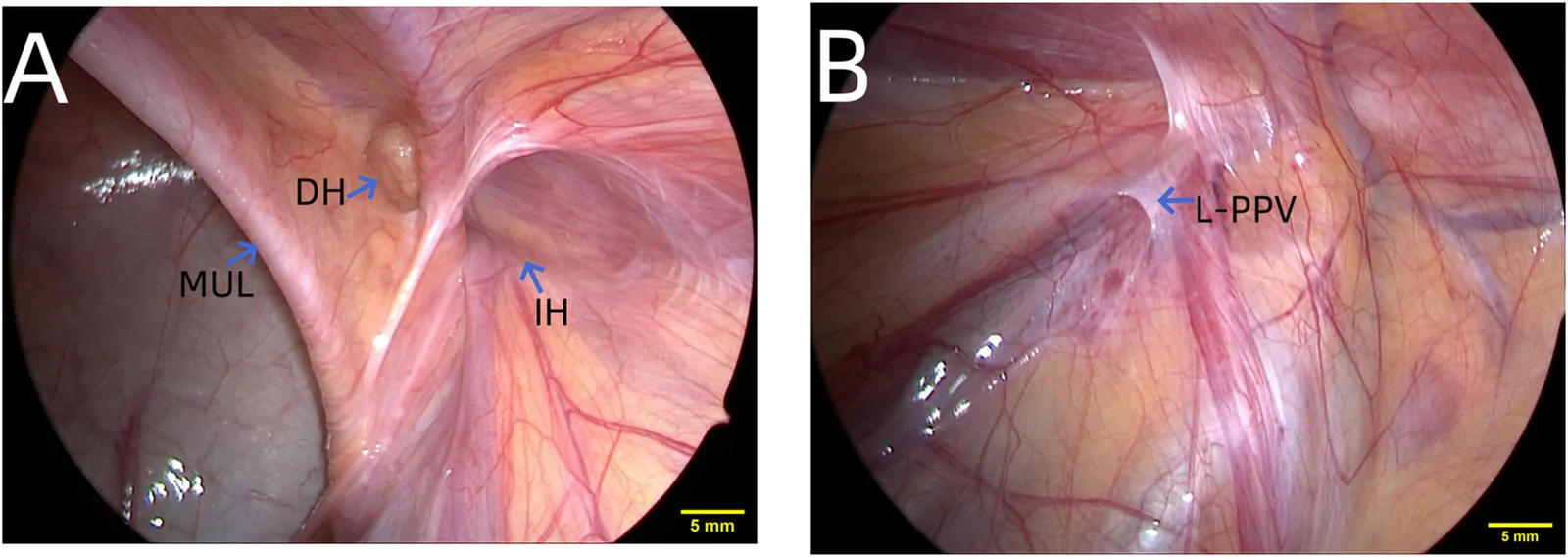
Laparoscopic views of the inguinal region. **(A)** Right-sided pantaloon hernia with coexisting direct (DH) and indirect (IH) inguinal hernia components, and a well-developed medial umbilical ligament (MUL). **(B)** Peritoneal fold covering the left internal inguinal ring (L-IIR), suggestive of a left patent processus vaginalis (L-PPV). DH, direct inguinal hernia; IH, indirect inguinal hernia; MUL, medial umbilical ligament; L-IIR, left internal inguinal ring; L-PPV, left patent processus vaginalis.

Under general anesthesia with a laryngeal mask airway, the patient was placed in the supine Trendelenburg position. A 5-mm incision was made at the superior umbilical rim, a trocar was inserted, and CO₂ pneumoperitoneum was established at a pressure of 9 mmHg. A 30° laparoscope was introduced. Laparoscopic inspection revealed a patent right internal inguinal ring measuring approximately 15 mm in diameter, and a separate defect of about 7 mm medial to the right inferior epigastric vessels, consistent with a diagnosis of pantaloon hernia (Figure 1A). On the left side, a peritoneal fold covered the internal inguinal ring (Figure 1B); during the subsequent right-sided repair, the fold was lifted using the double-hook hernia needle, revealing a patent processus vaginalis of approximately 3 mm in diameter. The right medial umbilical ligament was well-developed, measuring about 1.5 cm in width (the maximum perpendicular distance between the edge of the ligament and the abdominal wall with the patient in the supine position), with no significant weakness.

**Figure 2:**
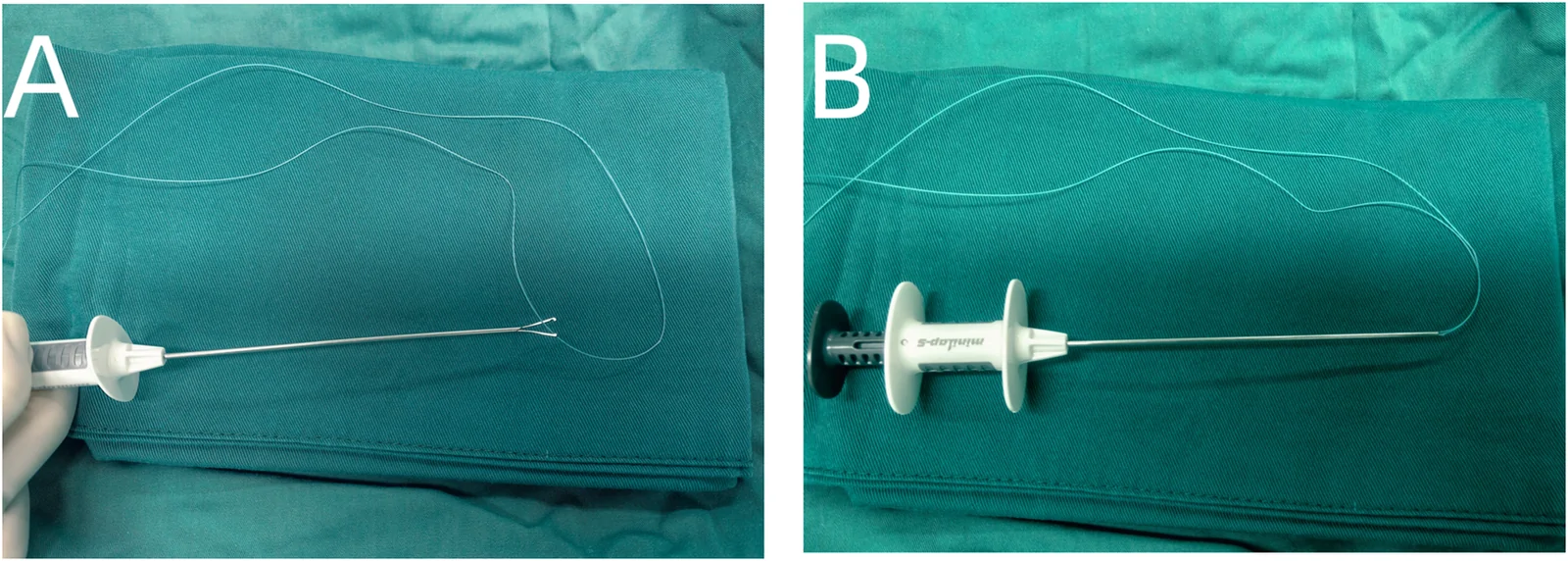
Double-hook hernia needle loaded with 3-0 non-absorbable suture **(A,B)**.

**Figure 3:**
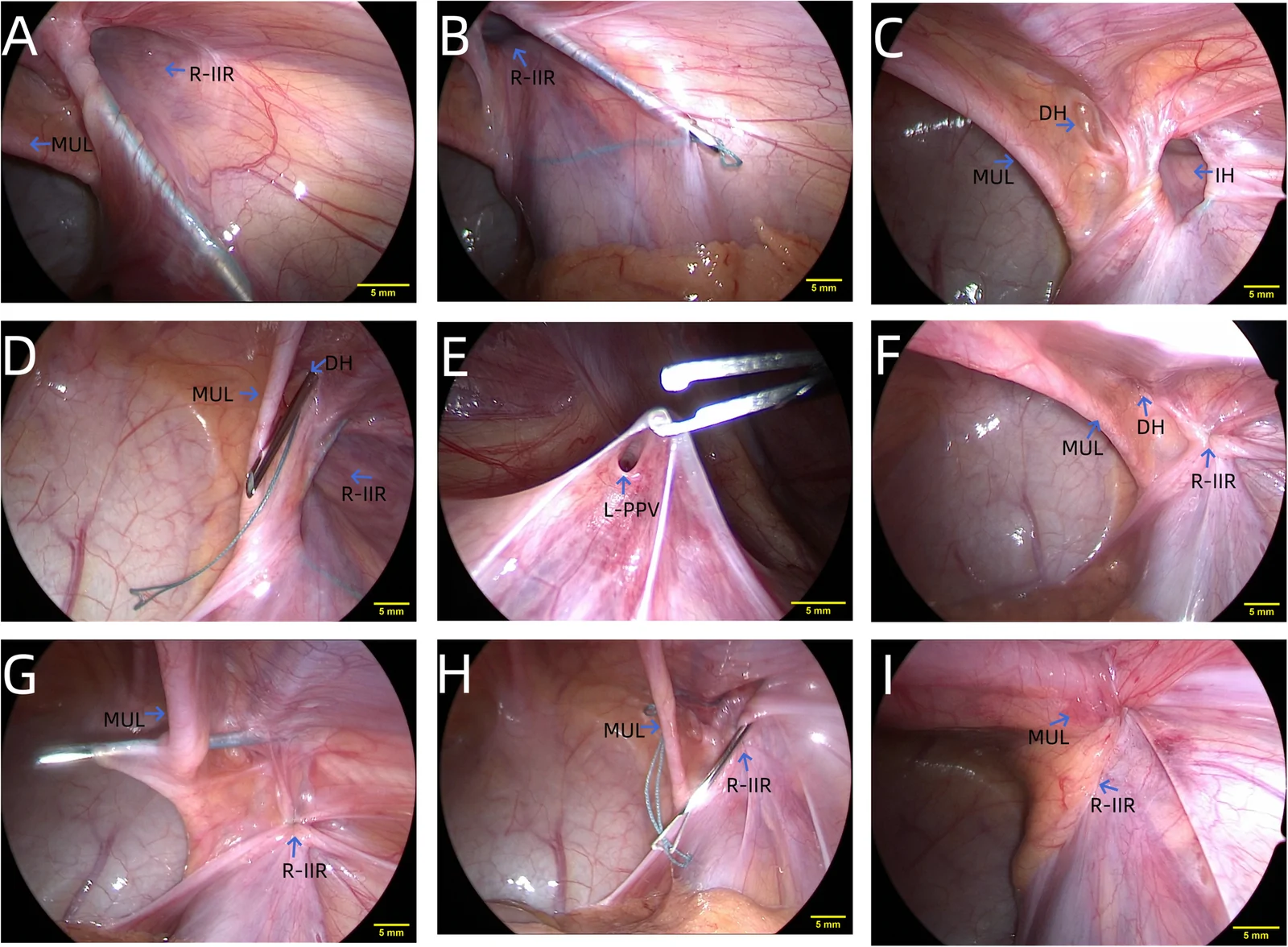
Laparoscopic repair of the right pantaloon hernia. **(A)** Hydrodissection along the medial aspect of the right internal inguinal ring (R-IIR) to separate the peritoneum from the spermatic vessels and vas deferens. **(B,C)** Retrieval of the preplaced suture through the original peritoneal puncture site. **(D)** Suture placement through the direct defect, with the needle withdrawn leaving the suture end in the peritoneal cavity. **(E)** Exploration of the L-IIR region using the hernia needle, revealing a L-PPV. **(F)** Appearance after successful high ligation of both the direct and indirect hernia sacs. **(G)** The hernia needle passed through the MUL at the superolateral aspect of the R-IIR. **(H)** Re-insertion of the needle laterally to retrieve the pre-placed suture. **(I)** Final view after completion of the reinforcement and coverage with the MUL. R-IIR, right internal inguinal ring.

**Figure 4:**
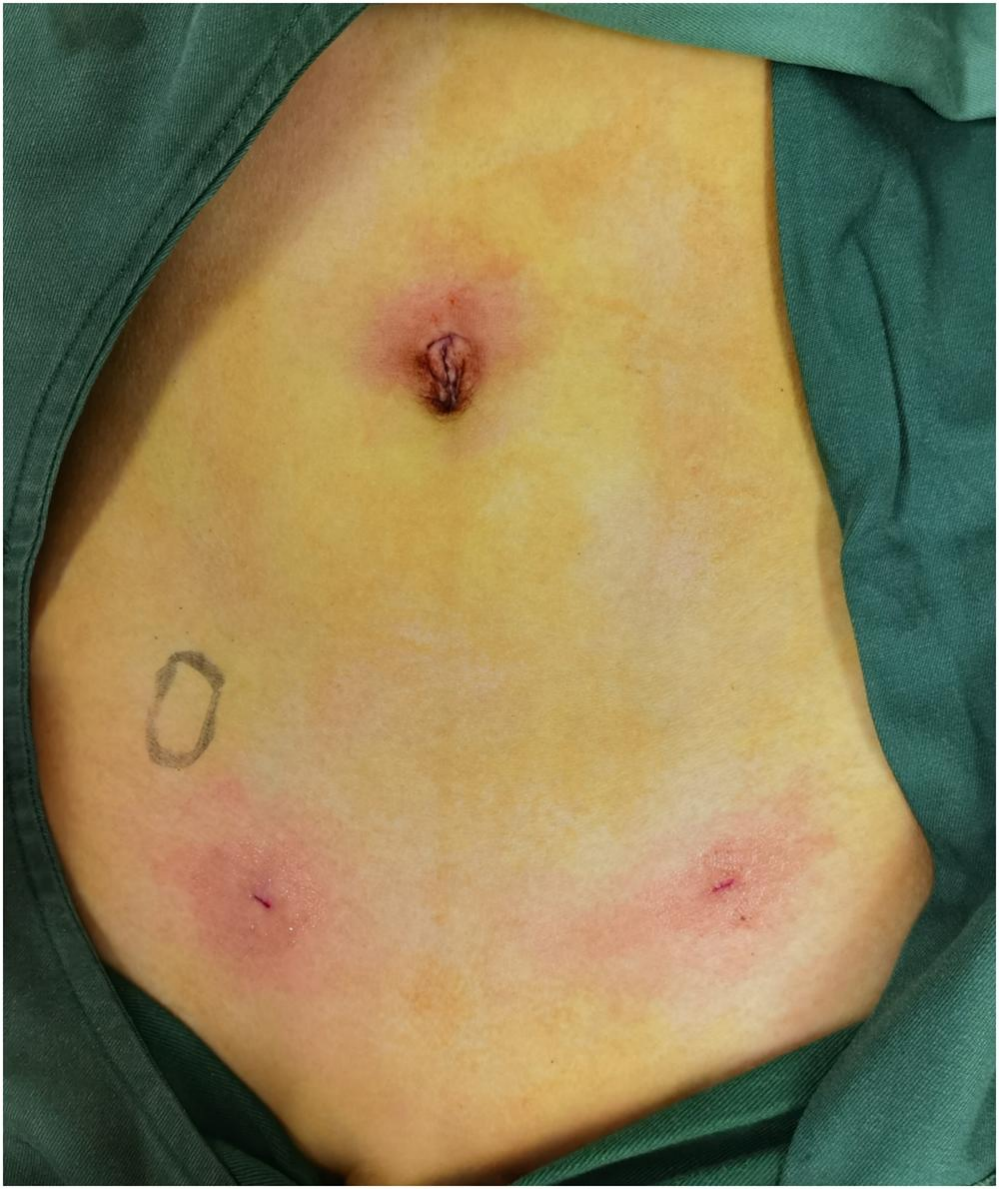
Postoperative appearance of the abdominal wall.

The procedure was carried out as follows:

Step 1: A 2-mm skin incision was made at the surface projection of the right internal inguinal ring. A double-hook hernia needle loaded with 3-0 non-absorbable suture was inserted (Figures 2A, B). Hydrodissection was used to separate the peritoneum from the spermatic vessels and vas deferens, preserving the gonadal structures. The needle was passed medially along the inner 180° of the internal inguinal ring, crossing the spermatic cord and vas deferens (Figure 3A), and entered the peritoneal cavity. The needle was withdrawn leaving the suture end in place, and then reinserted laterally through the original peritoneal puncture site to retrieve the pre-placed suture (Figures 3B, C).
Step 2: Through the same skin incision, the needle was advanced medially along the lateral margin of the direct defect, and the distal suture end was placed in the peritoneal cavity below the defect. After withdrawing the needle, it was reinserted along the medial margin of the direct defect, entering through the same peritoneal puncture site to retrieve the suture (Figure 3D). Prior to retrieval, the needle was advanced sufficiently to explore the left internal inguinal ring region; lifting the peritoneal fold confirmed a left patent processus vaginalis of about 3 mm (Figure 3E). The suture was retrieved extracorporeally, and after releasing pneumoperitoneum, the direct and indirect defect sutures were tightened separately and secured with double ligations under direct vision (post-ligation appearance in Figure 3F).
Step 3: A tunnel was created superolaterally through the same right skin incision. The needle was passed through the medial umbilical ligament at the superolateral aspect of the right internal inguinal ring (Figure 3G), leaving the suture end. It was then reinserted laterally to retrieve the pre-placed suture (Figure 3H). After releasing pneumoperitoneum, the suture was tied with a double ligation extracorporeally, completing the reinforcement and coverage with the medial umbilical ligament (Figure 3I).
Step 4: Through a 2-mm skin incision at the projection of the left internal inguinal ring, high ligation of the left patent processus vaginalis was performed using the same technique. After confirming no intra-abdominal injury or bleeding, the laparoscope was withdrawn, pneumoperitoneum was released, and the umbilical incision was closed (Figure 4).

The procedure was completed uneventfully, with a total operative time of 28 min and minimal blood loss (approximately 2 mL). No additional analgesic medication was required postoperatively. The patient was ambulatory at 6 h with minimal discomfort and was discharged on the following day. The family was advised to avoid strenuous activity, coughing, constipation, and heavy lifting for 3 months. Follow-up visits were scheduled at 1, 3, and 6 months postoperatively. At each visit, the groin region were examined for any bulging masses, induration, or tenderness. At 6-month follow-up, there was no inguinal pain, hematoma, recurrence, infection, or suture-related complications.

## Discussion

Direct inguinal hernia accounts for approximately 81.5% of rare hernias in children (2, 10). It may be congenital or acquired, resulting from weakness of the posterior wall of the inguinal canal that allows protrusion of abdominal contents through the fascia, rather than a single discrete defect (2, 11–13). Wright classified direct inguinal hernias into five types (6, 14): (1) diffuse weakness of the posterior wall without a significant direct sac; (2) combined direct and indirect hernias (pantaloon hernia); (3) isolated direct hernia without indirect component; (4) sliding direct hernia; and (5) massive indirect hernia with disruption of the posterior wall integrity. Pediatric direct hernias are more common on the right side and in males (1, 10); the present case of pantaloon hernia is consistent with these features. Pediatric pantaloon hernias constitute roughly 4.3% of “atypical” hernias (2), and clinical reports are limited. The etiology remains unclear but may be associated with congenital abdominal wall weakness or defects, prematurity, and chronic elevated intra-abdominal pressure (3, 11, 13, 15). Because the coexistence of direct and indirect components implies weakness of the posterior wall, simple ligation of the internal ring and the direct defect may be insufficient. Simultaneous reinforcement with the medial umbilical ligament can strengthen the posterior wall of the inguinal canal and reduce the risk of recurrence.

Esposito et al. recommended closing the direct defect with non-absorbable sutures and using the medial umbilical ligament as an autologous tissue patch for reinforcement (16). Intraoperative assessment of the thickness and development of the ligament is essential to determine the feasibility of this technique (6). If the ligament protrudes less than 1 cm from the peritoneum, reinforcement is not recommended because it may cause bladder displacement, excessive tension, and suprapubic pain during micturition (5). We observed a technical nuance: passing the needle through the medial umbilical ligament at the superolateral aspect of the internal ring and retrieving it laterally allows maximal mobilization of the ligament and increases the coverage area over the posterior wall. In cases of hypoplasia or absence of the ligament, alternative methods should be considered.

With the widespread use of laparoscopy, rare hernias in children are being identified more frequently (17). However, awareness of direct hernias remains limited among some clinicians. We recommend that all children with clinically diagnosed inguinal hernias undergo laparoscopic exploration to carefully inspect the groin. Even when an indirect hernia is confirmed, the Hesselbach triangle should be routinely examined with gentle pressure to rule out an occult direct defect. Koivusalo reported that four cases (3%) of suspected indirect recurrence were actually previously undiagnosed direct hernias (18). Furthermore, if no patent internal ring is found on the symptomatic side, meticulous examination for direct or femoral hernias is essential; missed diagnoses may lead to recurrence and reoperation. Importantly, the bladder should be emptied preoperatively in all cases to prevent a distended bladder from obscuring medial defects (e.g., direct or femoral hernias) (19). Selective restoration of abdominal wall muscle tone during anesthesia may also help the surgeon detect subtle weaknesses.

For a pantaloon hernia with coexisting direct and indirect components on the same side, Gödeke and Muensterer (2) recommended the Hoquet maneuver, which involves ligation of the inferior epigastric vessels during open or laparoscopic surgery to convert the two sacs into a single sac, which is then managed as a standard direct hernia. Ghosh et al. (3) reported a case of a 5-month-old premature male infant with a giant right inguinal hernia, in whom open exploration revealed a pantaloon hernia, and the defect was repaired with non-absorbable sutures to reinforce the floor of the inguinal canal. Bahadir et al. (1) reported a pantaloon hernia confirmed laparoscopically with combined medial and lateral defects, which was repaired by simple high ligation of both the direct and indirect components. Chin et al. (13) described a high-risk infant with extreme prematurity and prolonged positive-pressure ventilation who underwent three inguinal hernia repairs. During the second operation, bilateral pantaloon hernias were identified; the indirect components were managed with repeat high ligation of the internal ring, while the direct components were repaired using intracorporeal suturing reinforced with the median umbilical ligament. However, recurrence occurred due to continued positive-pressure ventilation, and the patient ultimately required reoperation for a right incarcerated hernia, which revealed a right indirect hernia and a left direct hernia. The right indirect hernia was repaired laparoscopically with high ligation, and the left direct hernia was managed with open Lichtenstein mesh repair.

In the present case, the patient had no prior history of inguinal hernia repair or connective tissue disorders. Since simple ligation of the direct hernia defect does not adequately address the underlying posterior wall weakness, we considered that reinforcement with autologous tissue would provide a more durable repair (10). We successfully performed transumbilical single-port laparoscopic high ligation of both the direct and indirect hernia sacs on the right side, combined with reinforcement using the ipsilateral medial umbilical ligament, without the need for additional working ports. The procedure was completed smoothly in a reasonable operative time, with an excellent postoperative outcome. This technique requires only a single 5-mm umbilical port, leaves minimal and cosmetically favorable scarring, and is relatively straightforward to learn and perform. It avoids extensive dissection and intra-abdominal suturing. To our knowledge, no such procedure has been previously reported for the management of pediatric pantaloon hernia. This case report provides a detailed step-by-step description of the surgical technique, accompanied by intraoperative images and technical nuances, thereby filling a gap in the current literature.

In a large series of 1,833 laparoscopic hernia repairs, Esposito et al. (4) reported contralateral patent processus vaginalis (PPV) in approximately 40% of cases. In traditional open surgery, 5% to 16% of patients undergoing unilateral repair may develop a metachronous contralateral inguinal hernia (MCIH), requiring repeat anesthesia and surgery (4, 20, 21). Current guidelines provide no definitive recommendation regarding exploration of an asymptomatic contralateral PPV (22). Schier et al. noted that because it remains unclear whether a small open processus vaginalis will eventually progress to a clinical hernia, they chose to close all open internal rings down to 2 mm in diameter, regardless of laterality, in an effort to eliminate the possibility of hernia development and reduce the risk of MCIH (12, 23). Laparoscopic exploration and prophylactic closure of a contralateral PPV have been shown to reduce the risk of MCIH (22, 24, 25). However, the presence of a PPV is a poor predictor of future MCIH (26). In our practice, we conducted a thorough preoperative discussion with the families of children with inguinal hernias, explaining the potential risks and benefits of contralateral exploration and management, including the possibility of MCIH and the need for reoperation. Most families agreed to laparoscopic exploration of the contralateral internal ring and concurrent repair of any identified abnormality. This represents a key advantage of the laparoscopic approach, which, with its magnified view and the use of hydrodissection during needle passage, minimizes the risk of injury to the vas deferens and spermatic vessels. For experienced laparoscopic surgeons, high ligation of a patent PPV adds little operative time and carries minimal risk of bleeding or scarring at the puncture sites. While contralateral exploration may expose the patient to unnecessary complications in some cases, it also avoids a potential second surgery and the associated risks of repeat anesthesia, which the U.S. Food and Drug Administration has warned may have deleterious effects on the developing brain (26). By closing the contralateral PPV at the initial operation, we can reduce the likelihood of MCIH, thereby avoiding the need for reoperation and the additional burden on both the child and the family.

In our patient, exploration of the left internal ring revealed a peritoneal fold; intraoperative lifting with the double-hook needle confirmed an underlying patent processus, which was immediately ligated. Our institution has previously encountered two cases where contralateral patency was inadequately evaluated during initial surgery, resulting in one case of contralateral communicating hydrocele and another of contralateral indirect hernia requiring reoperation. These experiences have reinforced the importance of meticulous contralateral exploration.

In this case, we used a double-hook hernia needle for puncture, suture manipulation, and tissue retraction. The instrument can extend double hooks to lift peritoneal folds without an additional grasping forceps, reducing the number of abdominal wall punctures and minimizing trauma. Using the same puncture site for repeated entries may reduce subcutaneous tissue injury and recurrence risk. However, when ligating the internal ring and direct defect, the needle should be withdrawn slowly to the preperitoneal level, not completely out to the subcutaneous tissue, before re-entering to retrieve the suture. This minimizes the amount of subcutaneous tissue incorporated in the ligature, thereby reducing the risk of ischemia, necrosis, and inflammation (27). In this case, multiple ligations (internal ring, direct defect, and umbilical ligament reinforcement) resulted in several knots. To minimize inflammatory reactions and recurrence risk, we performed double ligations in the extraperitoneal space, which reduced tension and decreased the risk of subcutaneous tissue strangulation and necrosis. At 6-month follow-up, no infection, pain, or recurrence was observed. Nevertheless, this follow-up period is relatively short, and longer-term outcomes regarding recurrence cannot yet be assessed. Extended follow-up with a larger cohort will be necessary to further evaluate the durability of this approach.

For pediatric direct inguinal hernias, we recommend individualized surgical strategies based on each patient's specific condition. According to the development of the medial umbilical ligament, defect size, and whether it is a recurrent hernia, different approaches should be considered. When the width of medial umbilical ligament is less than 1 cm or hypoplastic, reinforcement is not recommended (5). For large defects, recurrent hernias, or complex cases, open repair or mesh hernioplasty may be indicated to reduce recurrence (5, 13, 28). Some authors have reported anatomical preperitoneal repair with autologous tissue reinforcement (6). In our previous experience, two children with direct hernias with defects <1 cm and hypoplastic medial umbilical ligaments underwent laparoscopic suture repair via a preperitoneal approach, without ligament reinforcement, with no recurrence at 1-year follow-up. In the present case, because the medial umbilical ligament was well-developed, we performed laparoscopic high ligation plus ligament reinforcement, achieving good outcomes. Therefore, individualized treatment based on the specific anatomy and surgeon's expertise is advisable for pediatric direct inguinal hernias.

## Conclusion

Laparoscopy should be considered the preferred approach for pediatric inguinal hernia repair. It allows comprehensive evaluation of both groins, reduces the risk of missed rare hernias and metachronous hernias, and enables individualized treatment according to specific anatomical variants, thereby promoting better postoperative recovery.

## Data Availability

The datasets presented in this article are not readily available because of ethical and privacy restrictions. Requests to access the datasets should be directed to the corresponding author.
